# Pembrolizumab as first-line treatment for recurrent and metastatic head and neck cancer – real-world single-centre data

**DOI:** 10.2340/1651-226X.2025.42128

**Published:** 2025-01-28

**Authors:** Bogdana Patachi, Kristian H. Jensen, Anita Gothelf, Mogens Bernsdorf, Jeppe Friborg, Claus A. Kristensen

**Affiliations:** Department of Oncology, Copenhagen University Hospital – Rigshospitalet, Copenhagen, Denmark

**Keywords:** Immunotherapy, head and neck cancer, real-world data

## Abstract

**Background and purpose:**

The randomised clinical trial KEYNOTE-048 has demonstrated a significant increase in survival for patients with head and neck cancer treated with pembrolizumab with or without chemotherapy. The purpose of the present retrospective study was to investigate whether survival in a group of consecutive patients treated at our department was comparable to the results from KEYNOTE-048.

**Patients/material and methods:**

Seventy-six patients initiated treatment with pembrolizumab ± platinum/5-FU between July 2020 and May 2022. Baseline characteristics were collected, response rates and survival times were calculated and compared to those published from KEYNOTE-048.

**Results and Interpretation:**

Fifty-one percent of patients had locoregional recurrence and 47% had distant metastases. Median progression-free survival was 5.5 months, and median overall survival (OS) was 12.3 months in the total cohort. OS was significantly higher for patients with combined positive score (CPS) ≥20 (14.6 months) than for patients with CPS 1–19 (7.3 months) (*p* = 0.04). There was no significant difference in survival times between patients ± 65 years of age or between patients with locoregional disease versus distant metastases. In conclusion, the results from KEYNOTE-048 were corroborated in a consecutive cohort of patients treated at Rigshospitalet, Copenhagen, Denmark.

## Introduction

At the time of primary diagnosis, head and neck cancer is often present as a loco-regional disease sensitive to (chemo-)radiotherapy with a favourable prognosis [1]. In patients with local recurrence and/or distant metastatic spread (R/M), the prognosis was previously very poor and unaffected by systemic therapy [2, 3]. In 2008, a randomised controlled trial (RCT) demonstrated an increase in median OS from 7.4 to 10.1 months with the addition of cetuximab to platinum and 5-fluorouracil (5-FU) [4]. In KEYNOTE-048, a significant further OS benefit for pembrolizumab monotherapy was demonstrated for patients with a programmed death-ligand 1 (PD-L1) combined positive score (CPS) ≥ 20 (14.9 months vs. 10.7 months) and ≥1% (12.3 months vs. 10.3 months) compared to platinum, 5-FU, and cetuximab [5]. When combined with chemotherapy, the OS for patients in the CPS ≥ 20 group in the pembrolizumab arm was 14.7 months versus 11.0 months for platinum, 5-FU, and cetuximab, and 13.6 months versus 10.4 months among patients with CPS ≥ 1. In June 2020, based on these results, pembrolizumab was approved in Denmark as monotherapy or in combination with chemotherapy for patients with CPS ≥ 1.

Since RCT populations tend to systematically differ from patients treated in routine clinical practice, it is paramount to ensure that benefits observed in RCTs remain present in non-trial populations [6, 7]. Therefore, this study sought to assess the effect of pembrolizumab with or without chemotherapy in a real-world cohort of patients with PD-L1 (CPS) ≥ 1 and to compare these results to those of KEYNOTE-048.

## Materials and methods

This is a retrospective observational study, including patients treated with the first dose of pembrolizumab at Rigshospitalet, Copenhagen, Denmark between July 2020 and May 2022. The study was approved according to national regulations with approval no. p-2024-17060.

Data were collected from patient files, laboratory reports, pathology reports, and imaging reports. A PET/CT scan was usually available at baseline and CT-scans were performed q. 9–12 wks. during treatment. Patient and treatment characteristics included age, sex, ECOG performance status (PS), smoking status, disease status (locoregional recurrence vs. distant recurrence), primary tumour location, type of treatment (pembrolizumab or pembrolizumab ± platinum/5-FU), date of treatment initiation, duration of treatment, reason for discontinuation, and toxicity. Tumour characteristics included histology, PD-L1 (CPS) status evaluated by immunohistochemistry, p16 status, and initial staging according to the TNM criteria (UICC 8^th^ edition). Almost one quarter of patients (23%) would not have fulfilled the inclusion criteria of KEYNOTE-048, because they had nasopharyngeal, sino-nasal, or unknown primary tumours. Three patients were treated for parotid cancers of the lymphoepithelial carcinoma subtype. The recommended first line treatment for R/M nasopharyngeal carcinoma is cisplatin and gemcitabine but PD-1 inhibitors have also proven to be active [8], and pembrolizumab was found to be the best choice for treatment of the patients included in this study. In order to make this report of real-world experience as complete as possible, it was decided to include all patients treated according to the KEYNOTE-048 principles.

Baseline characteristics were summarised using descriptive statistics. The Mann–Whitney U and Chi-square tests (IBM SPSS Statistics) were used for comparison of tumour characteristics between subgroups. Survival times were calculated from the date of treatment initiation and subsequent time-to-event analyses were conducted using the Kaplan–Meier method. The log-rank method was used to assess the statistical significance of differences in survival distributions, with significance level of 0.05. The last follow-up date was 31 October 2023. All survival analyses were performed using R version 3.6.

## Results

Seventy-six patients were included. Baseline characteristics are presented in Table 1. Patients were treated with pembrolizumab 200 mg i.v. Q3W or 400 mg i.v. Q6W. Chemotherapy was given according to KEYNOTE-048 [5]. The median age was 65 years and the ratio of women to men was 1:3. Most patients (86%) were PS = 1. Oropharynx was the most frequent tumour subsite (32%), followed by the oral cavity (21%), hypopharynx (13%), and larynx (12%). There was no difference in PD-L1 CPS between patients with diagnoses included (median CPS = 20, range 1–100) versus not included (median CPS = 22.5, range 1–90, *p* = 0.97) in KEYNOTE-048.

**Table 1:** Pretreatment characteristics.

	All treated patients	KEYNOTE-048
Age (*N* = 76)	65 (58–73)* years	62 (56–68)* years
Sex (*N* = 76)	F: 19 (25%)	19%
	M: 57 (75%)	81%
ECOG/WHO PS (*N* = 73)	0: 8 (11%)	39%
	1: 63 (86%)	61%
	2: 2 (3%)	0%
Primary tumour	Oropharynx: 24 (32%)	39%
location (*N* = 76)	p16 + : 13 (54%)	54%
	p16 - : 11 (46%)	46%
	Oral cavity: 16 (21%)	28%
	Hypopharynx: 10 (13%)	14%
	Larynx: 9 (12%)	21%
	Nasopharynx: 8 (11%)	
	Sino-nasal: 3 (4%)	
	Unknown primary 3 (4%)	
	Parotid gland (LEC): 3 (4%)	
Oropharyngeal
p16 positive (*N* = 76)	13 (17%)	21%
PD-L1 (CPS) (*N* = 76)	1–19: 35 (46%)	48%
	≥ 20: 41 (54%)	52%
Disease status (*N* = 76)	Locoregional recurrence only: 39 (51%)	27%
	Metastatic: 36 (47%)	72%
	Newly diagnosed, non-metastatic: 1 (1%)	1%
Type of treatment (*N* = 76)	Pembrolizumab: 59 (78%)	
	Pembrolizumab + platinum/5-FU: 17 (22%)	

CPS: Combined positive score; LEC: Lymphoepithelial carcinoma; PS: Performance status.

* Interquartile range.

Fifty-four percent of oropharyngeal cancers (OPCs) were p16-positive and p16-positive OPCs constituted 17% of the total cohort. There was no significant difference in age (median 62 years vs. 65 years, *p* = 0.28) or PS (*p* = 0.31) between p16-positive and p16-negative OPCs and p16-positive patients were not significantly younger than other patients (median age 62 years vs. 66 years, *p* = 0.17). The same fraction of patients (23%) received chemotherapy in p16-positive and other patients. As expected, the treated patients were evenly distributed in the groups with PD-L1 CPS 1–19 and ≥20.

Approximately half (51%) of the patients had locoregional recurrence only, the other half had distant metastatic disease (47%) or were given palliative treatment with pembrolizumab in the primary setting (1%).

Fifty-nine out of the total population of 76 patients completed at least the first evaluation scan after four treatment cycles (12 weeks) and were thus available for response assessment. Twelve patients (20%) achieved an objective response, five (8%) with PR and seven (12%) with CR as best response. The response rate for patients treated with pembrolizumab monotherapy was 17% (8 of 47 patients) and 33% (4 of 12 patients) for patients treated with pembrolizumab and chemotherapy in combination. Median time to first response for all 59 patients was 81 days (range 69–279 days).

All patients except one had recurrent disease; primary treatment was surgery and radiotherapy ± concomitant weekly cisplatin (40 mg/m^2^) and some patients had surgery for locoregional recurrence before treatment with pembrolizumab.

Early recurrence after primary therapy [9] and lymphopenia [10] are poor prognostic factors. It is possible, that previous lymphadenectomy or radiotherapy towards neck nodes induces lymphopenia and consequently lower response probability. There was, however, no difference in blood lymphocyte level between patients with early (<180 days) recurrence versus later recurrences (0.99 × 10^9^/L vs. 1.15 × 10^9^/L, *p* = 0.294) and no difference in response rate between patients with (19%) and without (21%, *p* = 0.855) lymphopenia (<1.0 × 10^9^/L) at the time of initiation of pembrolizumab treatment.

**Figure 1:**
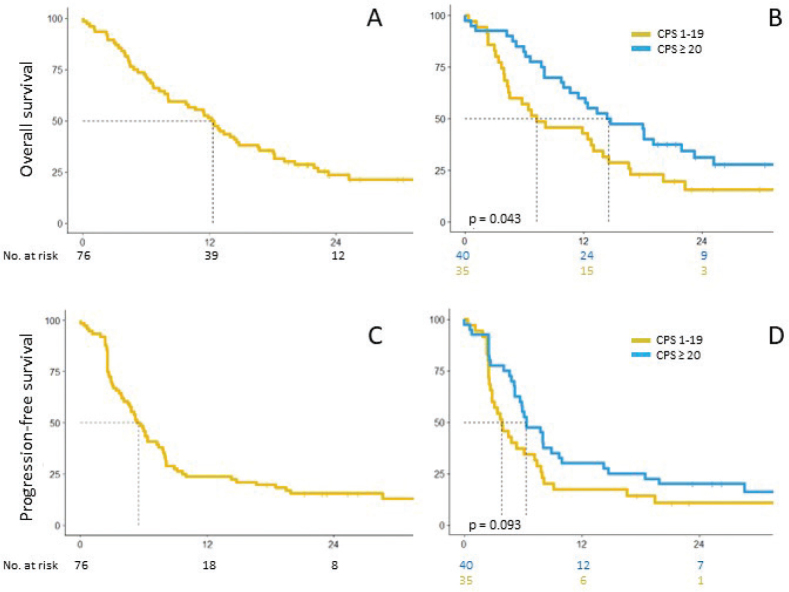
OS and PFS for the whole population (A and C) and divided in CPS subgroups (B and D).

Progression-free (PFS) and OS curves are shown in Figure 1. Median PFS and OS were 5.5 and 12.3 months, respectively, for the total cohort; 2-year PFS and OS was 16% and 24%, respectively (Figure 1C and 1A). Divided into subgroups, the median PFS for patients with CPS 1–19 was 3.9 and 6.4 months for patients with CPS ≥ 20 (*p* = 0.09) and OS was 7.3 and 14.6 months for the same two subgroups (*p* = 0.04). Two-year PFS and OS for patients with CPS 1–19 was 11% and 16% and for patients with CPS ≥ 20, PFS and OS was 20% and 31%, respectively (Figure 1B and 1D).

There were no significant differences in PFS or OS between age groups (±65 years) or patients with locoregional disease or distant metastases: Median PFS for patients with locoregional disease only and distant metastases was 6.1 and 4.5 months, with a median OS of 12.4 and 12.2 months, respectively. Patients older than 65 years had a median PFS of 5.4 months and median OS of 10.0 months; the corresponding survival times for patients under 65 years were 5.7 and 12.4 months, respectively.

Pembrolizumab-related adverse events occurred in 17 patients (22%); of these, three patients (4%) experienced grade 3+ events (pneumonitis, skin toxicity, and hypophysitis). Reported grade 1–2 toxicities were hypothyroidism, colitis, mucositis, nephritis, adrenal insufficiency, hepatitis, insulin-dependent diabetes mellitus, myositis, pancreatitis, polymyalgia, reversible encephalopathy syndrome, skin toxicity, thrombocytopenia, and thyroiditis. No treatment- related deaths occurred.

## Discussion

RCTs provide the most reliable documentation for effectiveness of new drugs. Patients included in RCTs are, however, highly selected, and the effect may not necessarily translate into effect in patients treated in daily clinical practice. Compared to results in RCTs, lower PFS and OS in the real-world setting has already been demonstrated in, for example, non-small cell lung cancer, likely due to relaxing of the criteria for treatment once the drug has been approved for use outside clinical trials [6].

As expected, the median age (65 years) was higher in this study than for those treated with pembrolizumab in KEYNOTE-048 (61–62 years). Also, the fraction of treated women was higher in the present cohort (25%) than in KEYNOTE-048 (19%). Although HPV-related OPC is the most common subtype of head and neck cancer in Denmark [11, 12], only 17% of this consecutive cohort of patients were p16-positive OPCs, probably reflecting the rarity of recurrence in these patients after initial treatment. The selection of patients with R/M disease for this study is probably also the reason for lack of significant age difference between patients with p16-positive OPCs and other patients, as it is seen in studies of baseline characteristics at primary diagnosis. In general, the patient population was comparable to the patients included in KEYNOTE-048, the major differences were in PS (11% in PS0 in this study vs. 39% in KEYNOTE-048) and in inclusion of other SCCs of the head and neck in our cohort. This expansion of the indication did not seem to affect the sensitivity to pembrolizumab, since there was no significant difference in CPS between the two diagnostic subgroups. Also, 51% of patients in our cohort had locoregional recurrence only versus 27% in KEYNOTE-048. This may be due to differences in surgical handling of loco-regional recurrence at different treatment centres, but there are no data to support this hypothesis.

Response rates for patients treated with pembrolizumab monotherapy (17%) and pembrolizumab + chemotherapy (33%) were quite comparable to the results from KEYNOTE-048: 19% and 36%, respectively, in the CPS 1 or more population.

In this study, the numbers were too small to separate patients in groups treated with and without chemotherapy and the patients were pooled in one group for survival analysis. Our survival data also corroborate the KEYNOTE-048 results; PFS in the group with CPS ≥ 1 was 5.5 months in our study and 3.2–5.0 months (w or w/o chemotherapy) in KEYNOTE-048, the corresponding numbers for OS are 12.3 months (present study) and 12.3/13.6 months (KEYNOTE-048 pembrolizumab ± chemotherapy). PD-L1 score (CPS) of 20 and above significantly increased the median OS from 7.3 to 14.6 months (*p* = 0.04) confirming the importance of PD-L1 score as a predictive factor for effect of pembrolizumab in head and neck cancer. Furthermore, as was the case in KEYNOTE-048, high PD-L1 scores did not seem to increase PFS, indicating that neither an objective response nor durable disease stabilisation may be necessary for effect of pembrolizumab on overall survival. There was no difference in neither PFS or OS between patients with locoregional disease only and patients with distant metastases or between patients ±65 years, indicating that also elderly patients should receive pembrolizumab when indicated.

In conclusion, response rates and survival data from KEYNOTE-048 were corroborated in a comparable, unselected group of patients in a real-life setting starting treatment consecutively over a 2-year period from July 2020 to May 2022.

## Data Availability

Data used in this study have been extracted from each patient’s medical record and are kept anonymised in a secured local database. Only approved investigators are allowed to access the data according to Danish regulations.
